# The Topical Uses of Tannic Acid

**Published:** 1882-11

**Authors:** 


### The Topical Uses of Tannic Acid.

Dr. G. A. Parsons, in the *Clinical Brief*, says that in all cases of cutaneous capillary hyperæmia, or the sub-cutaneous cellular transudations, the topical use of—

℞. Tannin, ℥ss
Glycerine, ℥j M

Triturate until perfect solution is effected, is very satisfactory.

In the first, or hyperæmic, stage of abscess, or risings, as they are familiarly called, the persistent application of a saturated solution of tannin in alcohol will, in a very large majority of cases, abort the pending abscess, and restore the part to a healthy condition without ever reaching the stage of suppuration.

In indolent ulcers, and all unhealthy sores, I have yet to find a remedy that meets the indication better than a triturated mixture of tannin and vaseline. In the different erythema, tannin is almost always the base of my prescriptions. In quite a number of cases of erysipelas, both simple and complicated, I have used tannin, either the glycerinic or alcoholic solution, with the most gratifying results. I never "paint erysipelas with iodine," as was formerly the practice. In sub-cutaneous cellular thickening and induration, the remedy of remedies is tano-iodine ointment made with—

℞. Tannin,
Iodine,
Vaseline, aa., q.s. M.

